# Comparing Nasal Patency Outcomes in Patients Undergoing Septoplasty with Radiofrequency Turbinate Reduction, Turbinectomy, or Valvuloplasty: A Prospective Cohort Study

**DOI:** 10.3390/medicina61091656

**Published:** 2025-09-11

**Authors:** Dejan Tomljenović, Lovro Grgurić, Mislav Knežević, Lucija Svetina, Goran Geber, Davor Vagić, Ageel Shibel, Andro Košec

**Affiliations:** 1 School of Dental Medicine, University of Zagreb, 10000 Zagreb, Croatia; dejan_tomljena@yahoo.com (D.T.); goran.geber1@gmail.com (G.G.); davorvagic1@gmail.com (D.V.); 2 Department of Otorhinolaryngology and Head and Neck Surgery, University Hospital Centre Sestre Milosrdnice, 10000 Zagreb, Croatia; 3 School of Medicine, University of Zagreb, 10000 Zagreb, Croatia; lovrogrguric@gmail.com (L.G.); dr.mknezevic@gmail.com (M.K.); 4 Department of Cardiac Surgery, University Hospital Center Zagreb, 10000 Zagreb, Croatia; lucijasv@gmail.com; 5 Faculty of Medicine, University of Novi Sad, 21000 Novi Sad, Serbia; shibel321@gmail.com

**Keywords:** nasal patency, rhinology, outcomes, septoplasty, turbinate reduction, nasal valve

## Abstract

*Introduction:* The aim of this study was to compare long-term postoperative nasal airflow and symptom improvement using both subjective and objective nasal patency outcomes after three common different types of surgical interventions in patients with nasal obstruction, aimed at reducing nasal obstruction. *Methods:* This is a single-center prospective cohort study on 105 patients performed in a tertiary rhinology center, who underwent one of three interventions: septoplasty with radiofrequency turbinate reduction, septoplasty with turbinectomy, or septoplasty with valvuloplasty, aimed at improving nasal patency. *Results:* All three groups showed statically significant postoperative improvement of the Nasal Obstruction Symptom Evaluation scale (NOSE) and Peak Nasal Inspiratory Flow measurement (PNIF) values that increased in time, with a markedly increased effect sustained during the six months of follow-up compared to baseline measurements (*p* = 0.001, Friedman’s two-way test). Intergroup analysis showed that septoplasty combined with radiofrequency turbinate reduction showed superior nasal patency results after three months (*p* = 0.001, Kruskal–Wallis test); however, all three groups had a similar result at the six-month follow-up. *Conclusions:* All surgical techniques in patients with anatomic nasal obstruction led to favorable postoperative subjective and objective nasal patency outcomes. However, the impact of any specific additional procedure alongside septoplasty may be limited in long-term follow-up.

## 1. Introduction

The nose is an organ with many functions and a complex structure. It is comprised of bone, cartilage, and soft tissue, while the anatomical interplay of the nasal septum and nasal turbinates regulates air humidification, heating, and regulation of airflow during nasal breathing [1]. The inferior nasal turbinate is the biggest, and its hypertrophy may cause nasal airway blockage and difficulty breathing. The internal nasal valve is an anatomical and physiological construct that is made up from the caudal nasal septum, upper lateral cartilages, and the inferior nasal turbinates, corresponding to the narrowest part of the nasal airway. It plays a vital role in regulating nasal airflow resistance, where its impact may be dynamic, static, or both [2].

The most common cause of nasal airway obstruction is septal deviation, which is present in 85% of patients with the described symptoms. Mild and severe turbinate hypertrophy exist in 71% and 6% of patients, while 34.4% of them have an insufficiency of the internal nasal valve [3]. Hypertrophy of inferior nasal turbinates is a common finding in patients with septal deviation, while compensatory hypertrophy of the nasal turbinate is present on the concave side of the deviated septum [4].

Nasal obstruction is a pervasive symptom encountered in otolaryngology, significantly impacting patients’ quality of life. While medical therapies are an initial approach, surgical intervention becomes necessary when conservative treatments fail. The aim of these procedures is to enhance nasal airflow and preserve the nose’s physiological functions, like filtering, warming, and moisturizing air. Septal deviation may be corrected solely with septoplasty or in combination with nasal turbinate reduction, with great variability in patient assessment and no concrete evidence on long-term postoperative nasal patency outcomes, since patients who have worse symptoms tend to show better postoperative results [5,6]. There is a variety of surgical treatment options for nasal turbinate hypertrophy if there is no response to medical therapy. Radiofrequency ablation, turbinoplasty, turbinectomy, and laser-assisted resection are the most widely used approaches. The most successful surgical technique often cited to improve postoperative nasal patency, mucosal recovery, and production of immunoglobulin A is submucosal resection. Radiofrequency ablation shows similar postoperative results compared to turbinoplasty. While inferior turbinate surgery results in favorable outcomes and continues to be routinely recommended as a treatment for turbinate hypertrophy not responsive to medical therapy, outcomes are still difficult to assess, as are the indications [7]. While endoscopic postoperative follow-up does show objective structural changes to anatomical structures affecting nasal patency, the physiological impact is difficult to assess accurately and may not be permanent in all patients [8].

The aim of this study was to compare postoperative nasal airflow and symptom improvement using both subjective and objective nasal patency outcomes after three different types of surgical interventions: septoplasty with turbinectomy, septoplasty with radiofrequency ablation of the nasal turbinates, or septoplasty with valvuloplasty.

## 2. Materials and Methods

**Figure 1:**
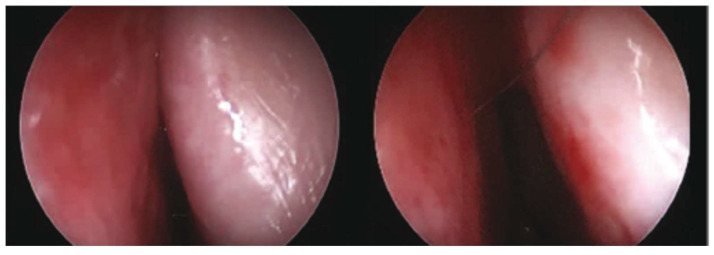
Typical pre- and post-operative endoscopic finding in patients undergoing septoplasty and additional nasal valve flaring suture placement. On the left, internal nasal valve collapse may be seen, while the right image shows significant internal nasal valve widening postoperatively.

This is a single-center prospective cohort study on 105 patients performed in a tertiary rhinology center, who underwent 1 of 3 intervention arms: septoplasty alongside 1 of 3 additional rhinologic procedures aimed at improving nasal patency (Figure 1).

The protocol was approved by the appropriate bioethical board adhering to the Ethical Principles for Medical Research Involving “Human Subjects”, adopted by the 18th World Medical Assembly, Helsinki, Finland, June 1964, and as amended most recently by the 64th World Medical Assembly, Fortaleza, Brazil, October 2013 (25 I-29-I I 13-23-03). The study was designed to comply with STROBE guidelines [9] and registered in the ClinicalTrials.org public database, NCT05948800.

The primary outcome measures were NOSE and PNIF measurements. The NOSE questionnaire score assessed subjective breathing quality through an internationally validated scale, consisting of 5 claims related to nasal obstruction, each divided into 4-point Likert scales (normal values 2.75–7, with scores >7 indicating clinically relevant nasal obstruction). Peak nasal inspiratory flow measurements were made using a calibrated instrument (PNIF, GM Instruments, with normal values ranging from 130 to 140 L/min for healthy young adults) [10,11,12]. PNIF is a noninvasive, physiologic measure indicating the peak nasal airflow in liters per minute achieved during maximal forced nasal inspiration. To reduce test–retest variability and sampling bias, three PNIF measurements were performed at every planned time interval, with the best measurement recorded for data analysis. The measurement was done by a fellowship-trained rhinologist. They did this by applying the device to occlude both nostrils and the mouth, after which subjects attempted to inhale exclusively through the nose with maximal effort.

The study recruited participants in the period from 24 July 2023 to 31 December 2024. A minimal study sample of 90 was calculated using G*Power version 3.1.9.7 (*t*-test, difference between two independent means), based on an effect size of 0.80, 80% study power, and an alpha error rate of 5%. The minimum significant difference in NOSE score was considered to be 0.8 points, with a reference value of 2.6 ± 1 points, based on published values for healthy individuals [10]. When looking at PNIF and sample size calculations as another primary outcome, a minimal study sample of 43 was calculated, with the minimum significant difference in PNIF score being 15 L/min, based on published values of 130 L/min and SD 35 L/min [11]. When testing for two predictors in a linear multiple regression model using G*Power, a total of 32 participants was calculated using a 0.35 effect size, 80% study power, and an alpha error rate of 5%, while an ANOVA model with 3 groups and a 0.40 effect size calculated a total sample size of 66, confirming adequate power of the study. Data collected included age, sex, and presence of allergy, where anthropometric and demographic variables were covariates and type of intervention was the dependent variable and primary outcome measure.

NOSE scores (Table 1) and peak inspiratory nasal flow measurements (PNIF) were performed at three time intervals: (a) preoperative measurement—basal values one day before surgery, both NOSE and PNIF, (b) three months postoperatively, both NOSE and PNIF, and (c) six months postoperatively, both NOSE and PNIF measurements.

**Figure 2:**
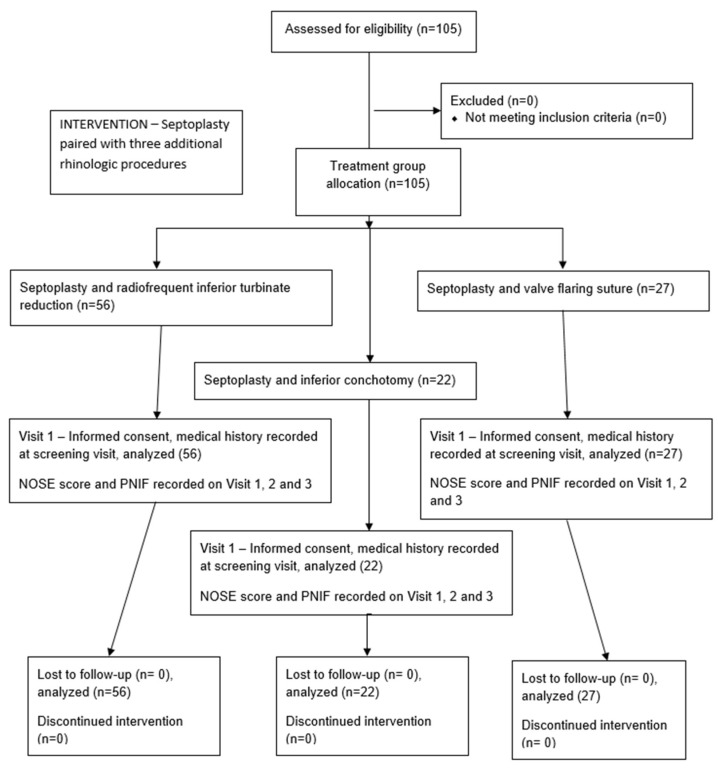
STROBE study flow diagram.

Inclusion criteria were met if patients were 18 years and above, without any disease or condition affecting nasal breathing apart from allergic rhinitis, septal deviation, or hypertrophy of the nasal turbinates. All patients were required to have both a deviated septum and inferior nasal turbinate hypertrophy as indications for surgery. The criteria required complete follow-up during the testing phases, complete documentation, written informed consent, and all questionnaire-related data to be complete. The patients were divided into three groups: those undergoing septoplasty with turbinectomy, those undergoing septoplasty with flaring sutures of the internal nasal valve, and those undergoing septoplasty with radiofrequency ablation of the inferior nasal turbinates. All of the surgeries were performed by two fellowship-trained rhinosurgeons, using a standardized technique in all three interventions (Figure 2).

The surgical technique of the intervention groups may be summarized as follows: In all groups, the septoplasty was performed through a submucosal septoplasty technique, creating incisions and elevating nasal mucosal flaps to expose and remove or reposition bent sections of the septal cartilage and bone, then closing the flaps to create a straighter septum. The stages included the hemitransfixion incision, elevation of the mucoperichondrial flaps, making incisions in the cartilage and bone, resecting the deviated portions, and repositioning the mucosal lining with dissolvable sutures and internal packing using Merocel splints (compressed polyvinyl alcohol (PVA) foam, Medtronic, Gallway, Ireland) to support the new septal structure.

As additional procedures, radiofrequency ablation (RFA) for the inferior nasal turbinates was performed bilaterally after septoplasty by delivering controlled heat to submucosal tissue using a thin probe (Celon ProBreath, Olympus, Hamburg, Germany). After administering local anesthesia, the RFA probe was inserted into the turbinates several times and energy was applied during several seconds, causing the tissue to shrink. The subtotal turbinectomy was performed bilaterally by using a microdebrider to reduce the size of the inferior turbinates by removing some of the soft tissue and bone, up to 50% of the intranasal volume to avoid adhesions and empty nose syndrome associated with over-resection. Flaring sutures of the internal nasal valve were placed during septoplasty by placing a horizontal mattress stitch to correct internal nasal valve collapse by pulling the upper lateral cartilages outwards, increasing the angle and area of the nasal valve for better airflow. It was passed through the upper lateral cartilage on one side of the nose, across the dorsum, and anchored to the other upper lateral cartilage, using the nasal septum as a fulcrum to spread the cartilages apart as the suture was tightened [12].

Patients with a history of psychological or mental illness, prior nasal surgery, or nasal polyposis were excluded from the study. Use of any medication related to airway management or nasal patency, such as decongestants or mucolytics, or nasal dilators to influence the external and internal nasal valve function, during the study period was not allowed.

Group allocation was performed by the leading physician and principal surgeon after discussing the surgical options with the patients. All patients were eligible for all three types of treatment based on their clinical findings and patient history. Since the type of surgery was unmasked and patients needed to have full disclosure of the planned intervention, voluntary cross-over between the groups was allowed.

### Statistical Analysis

Statistical analysis was performed depending on the normality of the distribution using the Kruskal–Wallis test, using an implemented Dunn’s pairwise post hoc test option to correct for multiple analyses, when comparing outcomes between the groups. Friedman’s two-way test was then performed to assess correlations between measurement intervals (the non-parametric alternative to the one-way ANOVA with repeated measures). Binary logistic regression was used to test whether presence of allergy was a confounding factor. All tests of statistical significance were performed using a two-sided 5% type I error rate. Statistical analysis was performed using MedCalc software (Version 11.2.1 © 1993–2010, MedCalc Software, bvba Software, Broekstraat 52, 9030 Mariakerke, Belgium) and SPSS (Version 22.0, 2013, IBM SPSS Statistics for Windows, IBM Corp., Armonk, NY, USA) using standard descriptive statistics and frequency tabulation, as indicated.

## 3. Results

The group of patients with septoplasty and radiofrequency inferior turbinate reduction consisted of 56 patients, the septoplasty and inferior turbinate resection included 22 patients, while the septoplasty and valve flaring suture group included 27 patients (Table 2).

When testing for preoperative differences among the three surgical groups, no differences in allergy presence, age, or sex were noted between the groups (*p* > 0.05, chi-square test). There were no differences in baseline NOSE score or PNIF values preoperatively (*p* > 0.05, Kruskal–Wallis test). The patients with presence of allergy were evenly distributed among the groups, without statistically significant differences in allocation (*p* > 0.05, chi-square test).

### 3.1. Postoperative Outcomes Regarding Time Intervals for All Groups

**Figure 3:**
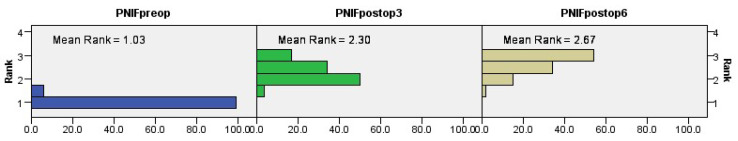
Friedman’s paired samples test showing the effect of surgery on PNIF in all three groups in the baseline, three-month, and six-month follow-up intervals (*p* = 0.001).

When analyzing all three groups and their postoperative breathing-related outcomes, Friedman’s two-way analysis of variance was used. The results showed that all three groups showed statically significant postoperative improvement of PNIF values that increased in time, with a markedly increased effect sustained during the 6 months of follow-up (*p* = 0.001, Friedman’s two-way test, Figure 3).

**Figure 4:**
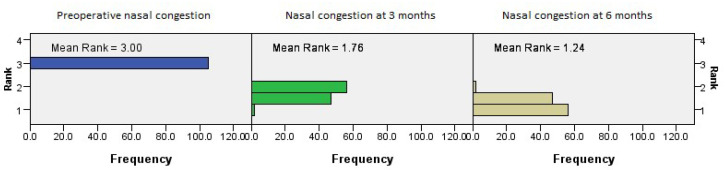
Friedman’s paired samples test showing the effect of surgery on nasal congestion in all three groups in the baseline, three-month, and six-month follow-up intervals (*p* = 0.001).

The postoperative outcomes with regard to NOSE score categories showed that the nasal congestion NOSE category continued to improve with time (*p* = 0.001, Friedman’s two-way test, Figure 4).

**Figure 5:**
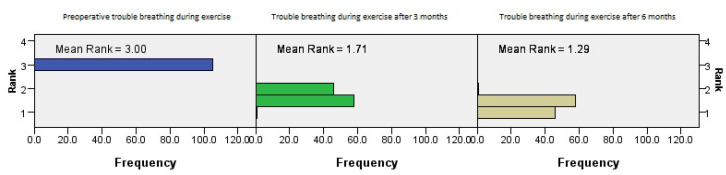
Friedman’s paired samples test showing the effect of surgery on trouble breathing during exercise in all three groups in the baseline, three-month, and six-month follow-up intervals (*p* = 0.001).

The postoperative outcomes of NOSE score category regarding trouble breathing during exercise showed continued improvement with time (*p* = 0.001, Friedman’s two-way test, Figure 5).

**Figure 6:**
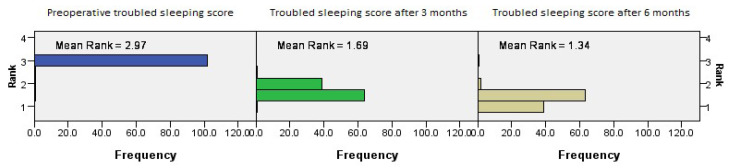
Friedman’s paired samples test showing the effect of surgery on trouble sleeping in all three groups in the baseline, three-month, and six-month follow-up intervals (*p* = 0.001).

The postoperative outcomes of NOSE score category regarding trouble sleeping showed continued improvement with time (*p* = 0.001, Friedman’s two-way test, Figure 6).

**Figure 7:**
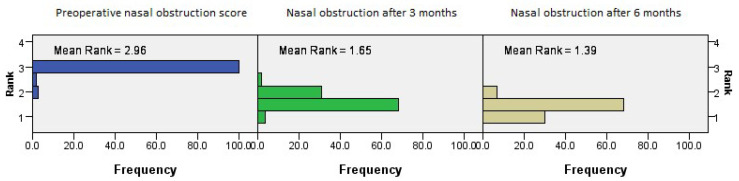
Friedman’s paired samples test showing the effect of surgery on nasal obstruction in all three groups in the baseline, three-month, and six-month follow-up intervals (*p* = 0.001).

The postoperative outcomes of NOSE score category regarding nasal obstruction showed continued improvement with time (*p* = 0.001, Friedman’s two-way test, Figure 7).

**Figure 8:**
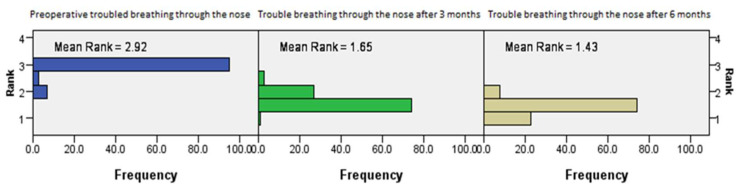
Friedman’s paired samples test showing the effect of surgery on trouble breathing through the nose in all three groups in the baseline, three-month, and six-month follow-up intervals (*p* = 0.001).

The postoperative outcomes of NOSE score category regarding trouble breathing through the nose showed continued improvement with time (*p* = 0.001, Friedman’s two-way test, Figure 8).

**Figure 9:**
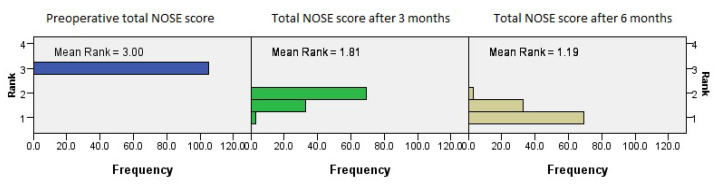
Friedman’s paired samples test showing the effect of surgery on total NOSE score values in all three groups in the baseline, three-month, and six-month follow-up intervals (*p* = 0.001).

The total NOSE scores reflect a continuous and stable improvement trend evident in all subcategories of the questionnaire, at both three months and six months (*p* = 0.001, Friedman’s two-way test, Figure 9).

### 3.2. Group-Specific Differences in Time-Related Postoperative Outcomes

**Figure 10:**
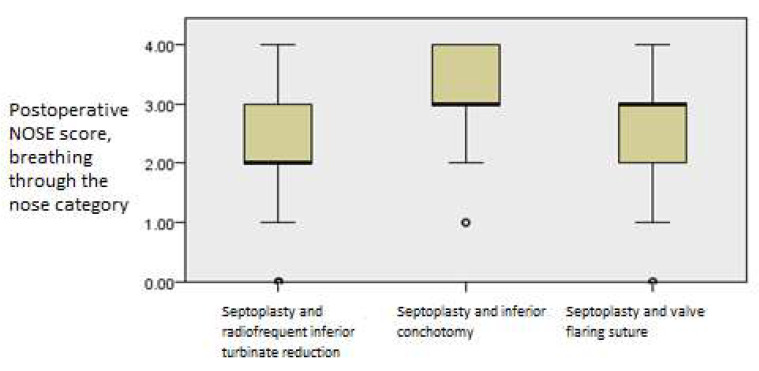
Independent samples Kruskal–Wallis test showing postoperative differences in NOSE values among the groups regarding trouble breathing through the nose at the first three-month interval (*p* = 0.001).

There were no differences in PNIF values among the groups after three or six months postoperatively (*p* = 0.097, Kruskal–Wallis test). With regard to postoperative NOSE values, trouble breathing through the nose was identified as significantly better in the septoplasty and radiofrequency inferior turbinate reduction group after the first three months (*p* = 0.009, Kruskal–Wallis test, Figure 10).

**Figure 11:**
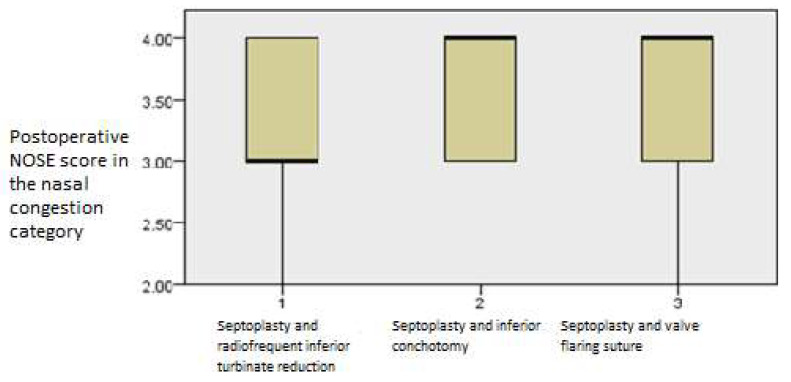
Independent samples Kruskal–Wallis test showing postoperative differences in NOSE values among the groups regarding the nasal congestion category at the first three-month interval (*p* = 0.001).

The same pattern was evident in the NOSE category regarding nasal congestion, showing that the septoplasty and RF inferior turbinate reduction group scored better than the other two groups *(p* = *0*.001, Kruskal–Wallis test, Figure 11).

**Figure 12:**
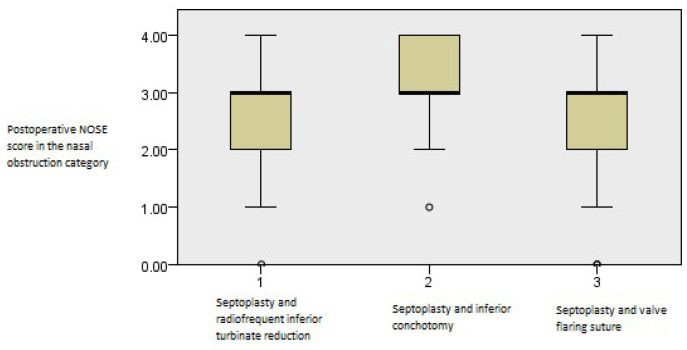
Independent samples Kruskal–Wallis test showing no postoperative differences in NOSE values among the groups regarding the nasal obstruction category at the first three-month interval (*p* > 0.05).

When nasal obstruction was tested, there were no significant differences among the groups at three months (*p* > 0.05, Kruskal–Wallis test, Figure 12).

**Figure 13:**
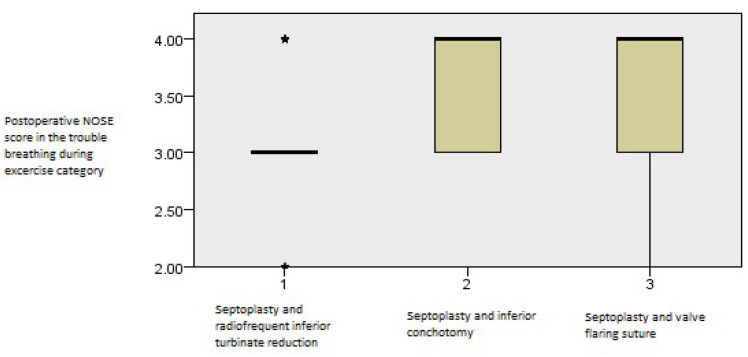
Independent samples Kruskal–Wallis test showing postoperative differences in NOSE values among the groups regarding the trouble breathing during exercise category at the first three-month interval (*p* = 0.001).

In the NOSE category regarding difficulty breathing during exercise, the septoplasty and RF inferior turbinate reduction group scored better than the other two groups (*p* = 0.001, Kruskal–Wallis test, Figure 13).

There were no differences in the trouble sleeping NOSE category between the groups at the first one-month interval (*p* = 0.058. Kruskal–Wallis test).

**Figure 14:**
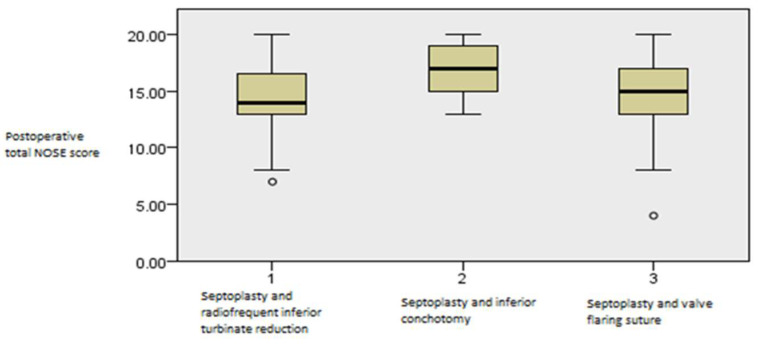
Independent samples Kruskal–Wallis test showing postoperative differences in total NOSE values among the groups in the first three months (*p* = 0.005).

When total NOSE scores were analyzed, the groups undergoing septoplasty alongside RF inferior turbinate reduction and flaring suture showed better NOSE scores compared to the septoplasty and inferior conchotomy group at the first one-month interval, and there were no differences compared to the septoplasty and nasal flaring valve group (*p* = 0.005, Kruskal–Wallis test, Figure 14).

When the third time interval after the six-month follow-up was tested, no statistically significant differences were found between the groups in any of the NOSE categories or PNIF measurements, suggesting that the procedure-associated variables were only relevant in the first several months after the surgery and did not have a lasting effect on nasal patency (*p* > 0.05, Kruskal–Wallis test).

To test for confounding factors, a binary logistic regression analysis was performed, with presence of allergy designated as the dependent variable, and there were no significant associations with the majority of NOSE score categories or PNIF values. The variables positively associated with presence of allergy had a lower preoperative PNIF value (*p* = 0.026, OR 4.928), which is to be expected, but were evenly distributed among the groups.

## 4. Discussion

Nasal airway obstruction is a complex issue faced by surgeons, as well as a potential concern after procedures, such as septoplasty and/or additional surgical interventions on the nasal turbinates and internal nasal valve. Septoplasty in patients with a septal deformity resulted in significant improvement in disease-specific quality of life, high patient satisfaction, and decreased medication use [13,14,15]. In case of a deviated septum accompanying additional factors (e.g., nasal turbinate size, allergy, nasal valve function, etc.), the outcomes have shown to be diverse. There is significant variation in the assessment, treatment, and outcomes of nasal airway obstruction and management in the published literature, which is why the aim of this study was to compare postoperative nasal airflow and symptom improvement using both subjective and objective outcome measures in patients after different types of surgical treatment.

Subjective patient-reported outcomes are widely recognized as crucial for evaluating surgical success in treating nasal obstruction. The Nasal Obstruction Symptom Evaluation (NOSE) scale is a validated questionnaire frequently used for this purpose, allowing quantification of subjective symptom severity [14], while PNIF measurements allow for a simple and objective comparator of nasal patency [11]. Objective measures like rhinomanometry and acoustic rhinometry quantify nasal flow resistance and permeability; however, most studies indicate a poor correlation between these objective measures and patients’ reported subjective improvement [16,17].

This study demonstrated that postoperative nasal patency issues, such as trouble breathing through the nose, nasal congestion, and difficulty breathing during exercise, were all shown to be significantly improved after septoplasty regardless of the additional accompanying procedure aimed at either the nasal turbinates or internal nasal valve. When nasal obstruction was tested, both the group with septoplasty and RF inferior turbinate reduction and that with septoplasty with valve flaring sutures showed statistically better NOSE scores than the septoplasty and turbinate resection group. Radiofrequency mucotomy, as a less invasive method for treating the nasal turbinates, has proven to be superior compared to subtotal mucotomy of the inferior nasal turbinates in suitable patients, likely due to reduced mucosal damage and improved postoperative healing [18].

While some perspectives suggest compensatory inferior turbinate hypertrophy might regress spontaneously after septoplasty, current evidence often favors addressing inferior turbinate hypertrophy in cases with a deviated nasal septum. Studies show that patients undergoing partial inferior turbinectomy in addition to septoplasty experience significantly greater symptomatic relief compared to those undergoing septoplasty alone. This holds true for symptoms like nasal congestion, nasal blockage, trouble breathing through the nose, trouble sleeping, and inability to get enough air during exertion [19,20].

One study indicated that for patients with inferior turbinate hypertrophy due to a deviated septum, septoplasty alone could lead to significant diminution of mucosal thickness on the concave side and increased thickness on the convex side, with associated high patient satisfaction. However, the initial hypertrophy is primarily mucosal, not bony, reinforcing the idea of a compensatory mechanism [21,22].

Radiofrequency turbinate reduction is an effective and popular technique due to its convenience and low morbidity. It induces submucosal tissue necrosis while sparing mucosal epithelium and turbinate bone, preserving mucociliary function. Studies confirm its efficacy in reducing nasal resistance and increasing nasal volume objectively, along with improving quality of life subjectively. Compared to partial inferior turbinectomy, published data suggest that both techniques achieve good clinical outcomes and improved nasal function. However, radiofrequency turbinate reduction appears to better preserve nasal physiology, as evidenced by histological analysis showing preserved ciliated epithelium and significantly shorter mucociliary transport time prolongation compared to turbinate resection, which can lead to diffused squamous metaplasia and loss of cilia [23,24]. Our results support these findings.

Interestingly, after the third time interval after the six-month follow-up was tested, no statistically significant differences were found between the groups in any of the NOSE categories or PNIF measurements, suggesting that the procedure-associated variables were only relevant in the first several months after the surgery and did not have a lasting effect on nasal patency.

Multiple studies consistently report significant subjective improvement in nasal symptoms following septoplasty, whether performed alone or in conjunction with turbinectomy. Symptoms like nasal obstruction, facial pain, sneezing, nasal pruritus, coryza, snoring, sleep disorders, and daytime drowsiness show marked improvement postoperatively. The most pronounced relief often occurs within the first seven postoperative days, continuing more gradually until the 60th day for most symptoms. Specifically, nasal obstruction improved in 94.4% of patients by the 60th postoperative day [21]. Another large study found a statistically and clinically significant reduction in NOSE scores at 1 month, with stability maintained at ≥6 months, suggesting that early subjective improvement is a reliable predictor of long-term nasal patency [22], consistent with our results.

There are several other reports that showed similar results to ours, who concluded that patients achieved statistically and clinically significant reductions in NOSE scores at 1 month, with no clinically significant differences in NOSE scores at ≥6 months, suggesting NOSE score stability between these postoperative time points [25,26]. However, very few studies compared septoplasty combined with internal nasal valve flaring sutures with the turbinate reduction and resection groups [27,28], adding a substantial degree of novelty in an otherwise very controversial indication area, centered on the critical question of when nasal valve issues necessitate specific surgical interventions. A recent review attempted to create a treatment algorithm on when to include nasal valve surgery alongside septoplasty but was limited by reliance on patient-reported outcomes, which introduce variability in assessing treatment success, which is an area where our results may help [26].

Limitations across the discussed studies include their mostly retrospective designs, variations in surgical techniques and follow-up durations, and the ongoing challenge of correlating objective and subjective outcome measures. Our study endeavored to eliminate these issues by employing a prospective cohort design, but still had possible confounding factors, such as small numbers, patients with allergic rhinitis, and surgical variability. Although after six months the three groups were comparable in terms of nasal breathing quality, the follow-up period remains short and may not adequately capture long-term stability of results, recurrence rates, or late complications. Being a single-center study, the surgical methods reflect the practice of one institution only, which limits generalizability. Future research could benefit from longer follow-up periods and larger sample sizes to solidify findings and further explore long-term outcomes and potential complications. Continuing efforts to refine surgical techniques, particularly those that are mucosal sparing, remain important to optimize patient comfort and long-term functional preservation.

## 5. Conclusions

An individualized approach to patients and the importance of considering various surgical techniques in patients with anatomic nasal obstruction led to favorable postoperative subjective and objective nasal patency outcomes. However, the impact of any specific additional procedure alongside septoplasty may be limited in long-term follow-up, supporting the use of the simplest and most mucosa-sparing procedures as the most effective surgical option.

## Figures and Tables

**Table 1:** NOSE questionnaire.

	Not a Problem	Very Mild Problem	Moderate Problem	Fairly Bad Problem	Severe Problem
Nasal congestion or stuffiness					
Nasal blockage or obstruction					
Trouble breathing through my nose					
Trouble sleeping					
Unable to get enough air through my nose during exercise or exertion					

**Table 2:** Summary of study variables and primary outcome data.

**All participants**	N = 105
Male	N = 74
Female Age in years	N = 31
Average ± SD	35.11 ± 12.11
**Treatment group allocation**	
Septoplasty and radiofrequency inferior turbinate reduction	N = 56
Septoplasty and inferior turbinate resection	N = 22
Septoplasty and internal nasal valve flaring suture	N = 27
Presence of allergic rhinitis symptoms	N = 26 (24.8%)
Preoperative PNIF value (L/min)	85.7 ± 24.9
Postoperative PNIF value at 3 months (L/min)	131.7 ± 31.8
Postoperative PNIF value at 6 months (L/min)	141.8 ± 24.1
**Baseline NOSE scores**	
Nasal congestion or stuffiness	3.4 ± 0.5
Nasal blockage or obstruction	2.7 ± 1
Trouble breathing through my nose	2.4 ± 1.1
Trouble sleeping	2.9 ± 0.9
Unable to get enough air through my nose during exercise or exertion	3.3 ± 0.5
Total NOSE score	14.6 ± 3.5
**NOSE scores at 3-month follow-up**	
Nasal congestion or stuffiness	0.7 ± 0.6
Nasal blockage or obstruction	0.4 ± 0.5
Trouble breathing through my nose	0.3 ± 0.5
Trouble sleeping	0.5 ± 0.5
Unable to get enough air through my nose during exercise or exertion	0.6 ± 0.6
Total NOSE score	2.5 ± 2.4
**NOSE scores at 6-month follow-up**	
Nasal congestion or stuffiness	0.2 ± 0.4
Nasal blockage or obstruction	0.2 ± 0.4
Trouble breathing through my nose	0.0 ± 0.2
Trouble sleeping	0.1 ± 0.3
Unable to get enough air through my nose during exercise or exertion	0.1 ± 0.3
Total NOSE score	0.6 ± 1.1

## Data Availability

Data are available from the corresponding author upon reasonable request.

## Public Involvement Statement

There was no public involvement in any aspect of this research.

## Guidelines and Standards Statement

This manuscript was drafted following the STROBE guidelines for observational studies.

## Abbreviations

The following abbreviations are used in this manuscript:

NOSE	Nasal Obstruction Symptom Evaluation
PNIF	Peak Nasal Inspiratory Flow
